# Influenza Vaccination Reduces Dementia Risk in Chronic Kidney Disease Patients: A Population-Based Cohort Study

**DOI:** 10.1097/MD.0000000000002868

**Published:** 2016-03-07

**Authors:** Ju-Chi Liu, Yi-Ping Hsu, Pai-Feng Kao, Wen-Rui Hao, Shing-Hwa Liu, Chao-Feng Lin, Li-Chin Sung, Szu-Yuan Wu

**Affiliations:** From the Division of Cardiovascular Medicine (J-CL, Y-PH, P-FK, W-RH, C-FL, L-CS), Department of Internal Medicine, Shuang Ho Hospital, Taipei Medical University, New Taipei City; Institute of Toxicology (S-HL, S-YW), College of Medicine, National Taiwan University; Department of Radiation Oncology (S-YW), Wan Fang Hospital; Department of Internal Medicine (J-CL, P-FK, S-YW), School of Medicine, College of Medicine, Taipei Medical University, Taipei; and Department of Biotechnology (S-YW), Hungkuang University, Taichung, Taiwan.

## Abstract

Taiwan has the highest prevalence of chronic kidney disease (CKD) worldwide. CKD, a manifestation of vascular diseases, is associated with a high risk of dementia. Here, we estimated the association between influenza vaccination and dementia risk in patients with CKD.

Data from the National Health Insurance Research Database of Taiwan were used in this study. The study cohort included all patients diagnosed with CKD (according to International Classification of Disease, Ninth Revision, Clinical Modification codes) at healthcare facilities in Taiwan (n = 32,844) from January 1, 2000, to December 31, 2007. Each patient was followed up to assess dementia risk or protective factors: demographic characteristics of age and sex; comorbidities of diabetes, hypertension, dyslipidemia, cerebrovascular diseases, parkinsonism, epilepsy, substance and alcohol use disorders, mood disorder, anxiety disorder, psychotic disorder, and sleep disorder; urbanization level; monthly income; and statin, metformin, aspirin, and angiotensin-converting enzyme inhibitor (ACEI) use. A propensity score was derived using a logistic regression model for estimating the effect of vaccination by accounting for covariates that predict receiving the intervention (vaccine). A time-dependent Cox proportional hazard model was used to calculate the hazard ratios (HRs) of dementia among vaccinated and unvaccinated CKD patients.

The study population comprised 11,943 eligible patients with CKD; 5745 (48%) received influenza vaccination and the remaining 6198 (52%) did not. The adjusted HRs (aHRs) of dementia decreased in vaccinated patients compared with those in unvaccinated patients (influenza season, noninfluenza season, and all seasons: aHRs = 0.68, 0.58, and 0.64; *P* < 0.0001, *P* < 0.0001, and *P* < 0.0001, respectively). In the sensitivity analysis, adjustments were made to estimate the association of age and sex; diabetes, dyslipidemia, hypertension, cerebrovascular diseases, anxiety disorder; and statin, metformin, ACEI, and aspirin use with the incidence of dementia in various models. A stronger protective effect against dementia risk was demonstrated during the noninfluenza season. Regardless of comorbidities or drug use, influenza vaccination was an independent protective factor and dose-dependently reduced the risk of dementia in CKD patients.

Influenza vaccination exerts dose–response and synergistic protective effects against dementia in CKD patients with dementia risk factors by reducing the incidence of dementia.

## INTRODUCTION

Chronic kidney disease (CKD) is associated with a high risk of cardiovascular diseases, end-stage renal disease (ESRD), infection, and malignancy and high mortality.^1^ Taiwan has the highest prevalence of ESRD worldwide.^2^ A large Taiwanese cohort study demonstrated a high prevalence of CKD (11.9%) in adults and an even higher prevalence (37.2%) among the elderly population.^3^ Wen et al demonstrated that patients with CKD had 83% higher mortality for all causes and 100% higher mortality for cardiovascular diseases.^3^ A moderate reduction in the glomerular filtration rate (GFR), defined as a serum creatinine level ≥1.5 mg/dL (133 μmol/L) for men and ≥1.3 mg/dL (115 μmol/L) for women, is associated with accelerated cognitive decline and increased dementia risk in elderly people.^4–6^ Renal impairment is associated with vascular dementia in individual with good to excellent health. Moreover, faster GFR decline is associated with more significant cognitive decline.^7^ These observations are consistent with accumulated data showing an increase in the risk of coronary heart disease in patients with mild to moderate CKD. Hence, CKD is considered a manifestation of vascular diseases.

Patients with CKD have a 100% higher risk of cardiovascular diseases, and many studies have shown that cardiovascular diseases are associated with dementia.^8–11^ The incidence of dementia among Taiwanese people aged 65 years and older increased from 4.1% in 1980 to 10.2% in 2007. A study of the population of Taiwan revealed that the prevalence of dementia was ∼1.7% to 4.3% among elderly people.^12^ In Taiwan, the high prevalence of CKD may be correlated with dementia. Establishing health policies for reducing dementia risk in patients with CKD is crucial for lowering the societal burden and the care cost.

The influenza vaccination policy in Taiwan has gradually expanded from high-risk populations to key spreaders to further reduce the number of influenza cases and deaths. Patients with CKD are considered a high-risk population for influenza. Hence, the government announces the priority groups for vaccinations every year on the basis of the recommendations of the Advisory Committee on Immunization Practices (ACIP).^13^ Here, we estimated the association between influenza vaccination and dementia risk in patients with CKD to determine whether influenza vaccination reduces dementia risk in such patients.

### Patients And Methods

The National Health Insurance (NHI) program, which was established in 1995, currently provides comprehensive health insurance coverage to 98% of >23 million people. In this study, data from the National Health Insurance Research Database (NHIRD) were used. No statistically significant differences were observed in age, sex, or healthcare costs between the sample group and all enrollees. According to the Taiwan Center for Disease Control, the influenza season is defined as the interval from October to March. Data in the NHIRD that could be used to identify patients or care providers, including medical institutions and physicians, are scrambled before being sent to the National Health Research Institutes for database construction and are further scrambled before being released to researchers. Theoretically, querying the data alone to identify people at any level by using this database is impractical. All researchers using the NHIRD and the data subsets must sign a written agreement declaring that they have no intention of attempting to obtain information that could potentially violate the privacy of patients or care providers.^14^

**FIGURE 1:**
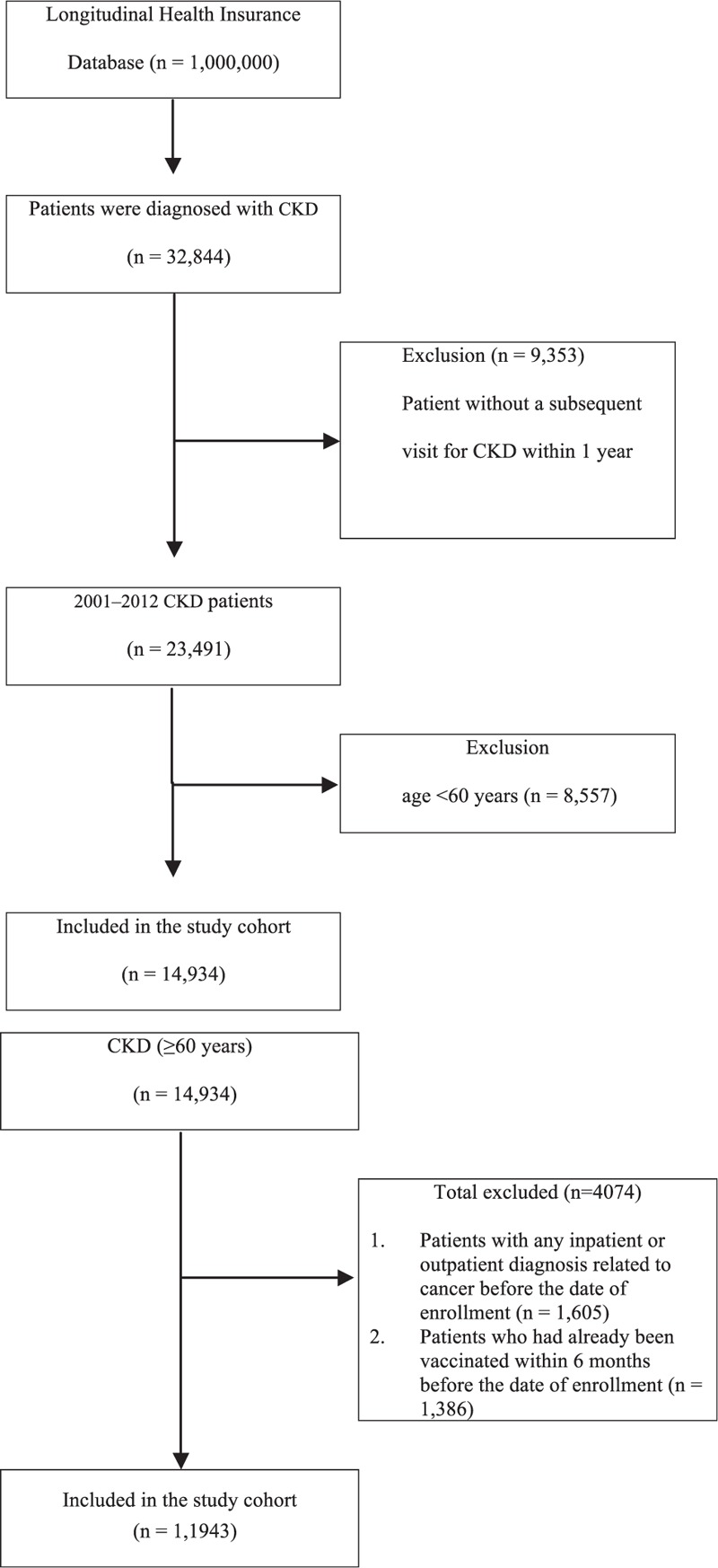
Patient selection flowchart.

The study cohort comprised all patients diagnosed with CKD (according to International Classification of Diseases, Ninth Revision, Clinical Modification [ICD-9-CM] codes) at healthcare facilities in Taiwan (n = 32,844) from January 1, 2000, to December 31, 2007. We excluded all patients without a subsequent outpatient visit, emergency department visit, or inpatient hospitalization for CKD within 12 months of the first presentation (n = 9535), because they were considered to not have CKD (Figure 1). We also excluded 27,276 patients who were <60 years (n = 8557) and had a history of any inpatient or outpatient diagnosis related to cancer before the enrollment date (n = 1605) or those vaccinated within 6 months before the enrollment date (n = 1386).

In Taiwan, influenza vaccination has been free of charge and recommended for high-risk adults aged ≥50 years (i.e., those with type 2 diabetes, chronic liver infection or liver cirrhosis, cardiovascular diseases, or chronic pulmonary diseases) since 1998 and for all adults aged >65 years since 2001. The vaccination status was identified using the ICD-9-CM code V048 and/or the use of vaccine (confirmed by drug codes).^15^ Our final study cohort contained 11,943 patients diagnosed with CKD in Taiwan >8-year period; 5745 were vaccinated against influenza and 6198 were not. Each patient was followed up to assess the risk of dementia or protective factors: the demographic characteristics of age and sex; the comorbidities of diabetes, hypertension, dyslipidemia, cerebrovascular diseases, parkinsonism, epilepsy, substance- and alcohol-use disorders, mood disorder, anxiety disorder, psychotic disorder, and sleep disorder; urbanization level; monthly income; and statin, metformin, aspirin, and angiotensin-converting enzyme inhibitor (ACEI) use. A propensity score (PS) was derived using a logistic regression model to estimate the effect of vaccination by accounting for the covariates predicting receiving the intervention (vaccine). This method is used in observational studies to reduce selection bias.^16^ The covariates in the main model were adjusted for PSs of age, sex, comorbidities, urbanization level (urban, suburban, and rural), monthly income (0; NT$1–NT$21,000; NT$21,000–NT$33,300; and ≥NT$33,301; NT$ represents new Taiwan dollars) (Table 2). The endpoint was the incidence of dementia (ICD-9-CM codes 331.0, 290.4, and 290.0–290.3) in vaccinated or unvaccinated patients with a subsequent outpatient visit, emergency department visit, or inpatient hospitalization for dementia within 12 months, and unvaccinated patients served as the reference arm. Because the protective effect of vaccination is specific to the influenza season, evaluating the noninfluenza season could indicate the possible contribution of bias to the estimates observed during the influenza season. The relationship between the seasonal effect of vaccination and dementia risk was also analyzed. The cumulative incidence of dementia in vaccinated and unvaccinated CKD patients was estimated using the Kaplan–Meier method. To examine the effect of the total number of vaccinations on the cumulative incidence of dementia, we categorized patients into 4 groups according to the vaccination status: unvaccinated and 1, 2 to 3, and ≥4 total vaccinations.

A time-dependent Cox proportional hazard model was used to calculate the hazard ratios (HRs) of dementia among vaccinated and unvaccinated patients with CKD. The HRs were adjusted for age, sex, baseline comorbidities, and metformin, statin, aspirin, and ACEI use in the multivariate analysis. A stratified analysis was conducted to evaluate the effect of vaccination on age and sex (Table 2). All analyses were conducted using SAS software, Version 9.3 (SAS, Cary, NC); 2-tailed *P* < 0.05 was considered significant. In sensitivity analyses, external adjustments improve the understanding of the effects of drugs and other covariates in epidemiologic database studies.^17^ Hence, in the sensitivity analysis, adjustments were made to estimate the association of age and sex; diabetes, dyslipidemia, hypertension, cerebrovascular diseases, and anxiety disorder; and statin, metformin, ACEI, and aspirin use with the incidence of dementia in different models. The models stratified by different seasons were adjusted for covariates in the main model and each additional covariate (Tables 3–5).

## RESULTS

The study cohort comprised 11,943 patients, of whom 5745 (48%) received influenza vaccination and the remaining 6198 (52%) did not (Table 1). The total follow-up duration was 17,234.8 and 35,899.1 person-years for unvaccinated and vaccinated patients, respectively. Compared with vaccinated patients, unvaccinated patients exhibited a higher prevalence of preexisting medical comorbidities including diabetes (*P* < 0.001), cerebrovascular diseases (*P* < 0.001), hypertension (*P* < 0.001), dyslipidemia (*P* < 0.001), substance- and alcohol-use disorders (*P* = 0.037), anxiety disorder (*P* < 0.001), and sleep disorder (*P* < 0.001). By contrast, vaccinated patients exhibited a higher prevalence of comorbidities such as parkinsonism (*P* < 0.001), mood disorder (*P* < 0.001), and psychotic disorder (*P* = 0.018). In addition, significant differences were observed in the distributions of age, monthly income, and urbanization level as well as statin, aspirin, ACEI, and metformin use between vaccinated and unvaccinated patients (Table 1). A higher proportion of unvaccinated patients used statin, metformin, ACEI, and aspirin for < 365 days; however, most vaccinated patients used these drugs for >365 days. A lower proportion of vaccinated patients had a monthly income of ≥NT$33,301 and resided in urban areas. Table 2 shows the risk of dementia among the unvaccinated and vaccinated patients in the study cohort. After adjustment for age, sex, comorbidities, urbanization level, and monthly income, we analyzed the PS. The adjusted HRs (aHRs) of dementia decreased in vaccinated patients compared with those in unvaccinated patients (influenza season, noninfluenza season, and all seasons: aHRs = 0.68, 0.58, and 0.64; *P* < 0.0001, *P* < 0.0001, and *P* < 0.0001, respectively). The stratified analysis showed that the aHRs still significantly decreased in vaccinated patients, particularly in patients aged ≥70 years, regardless of sex. During the noninfluenza season, the aHRs decreased regardless of age or sex, except for the 60 to 69 age group, whose sample size was relatively small compared with that of the other subgroups (Table 2).

**TABLE 1:**
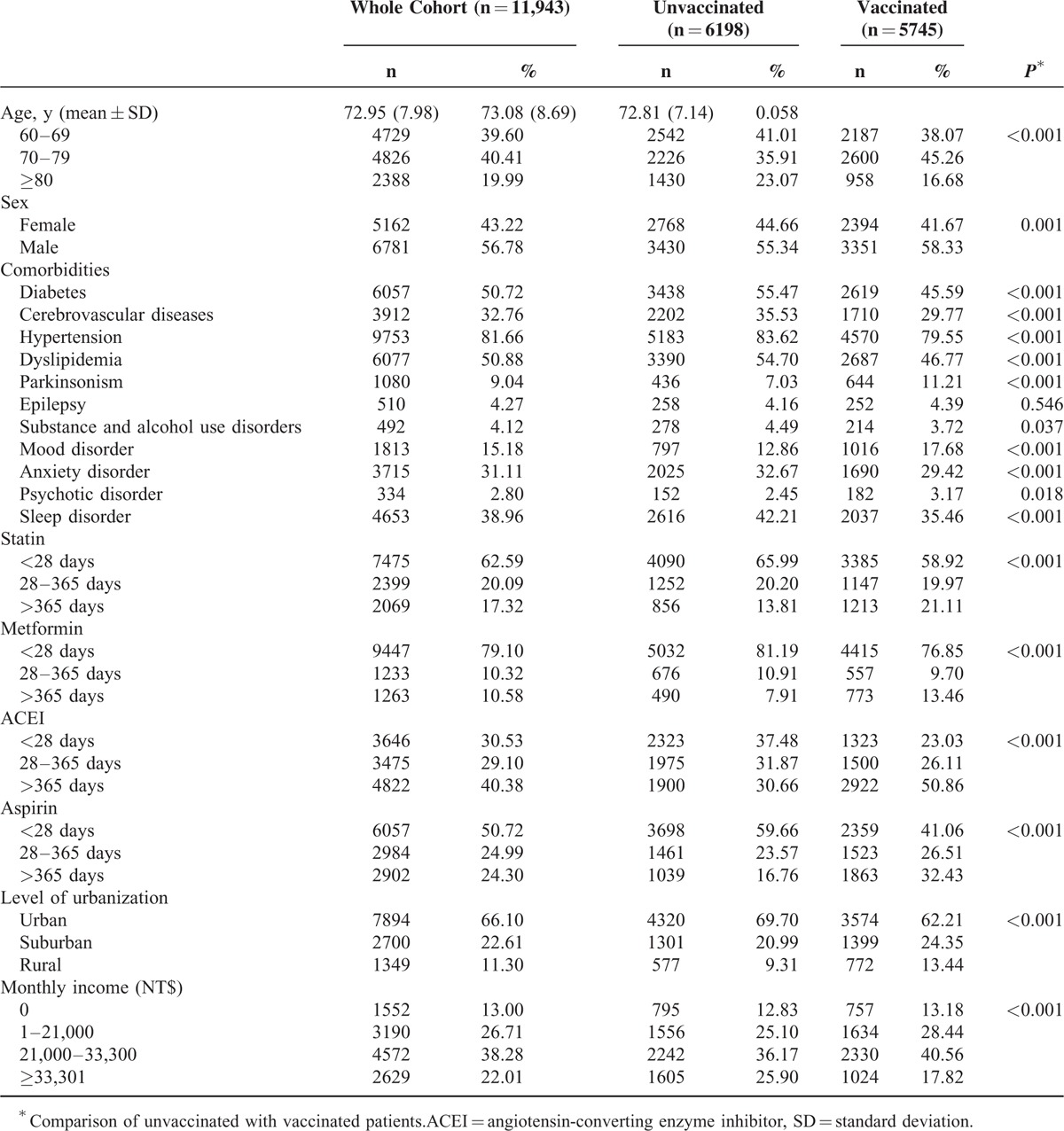
Characteristics of the Study Population

**TABLE 2:**
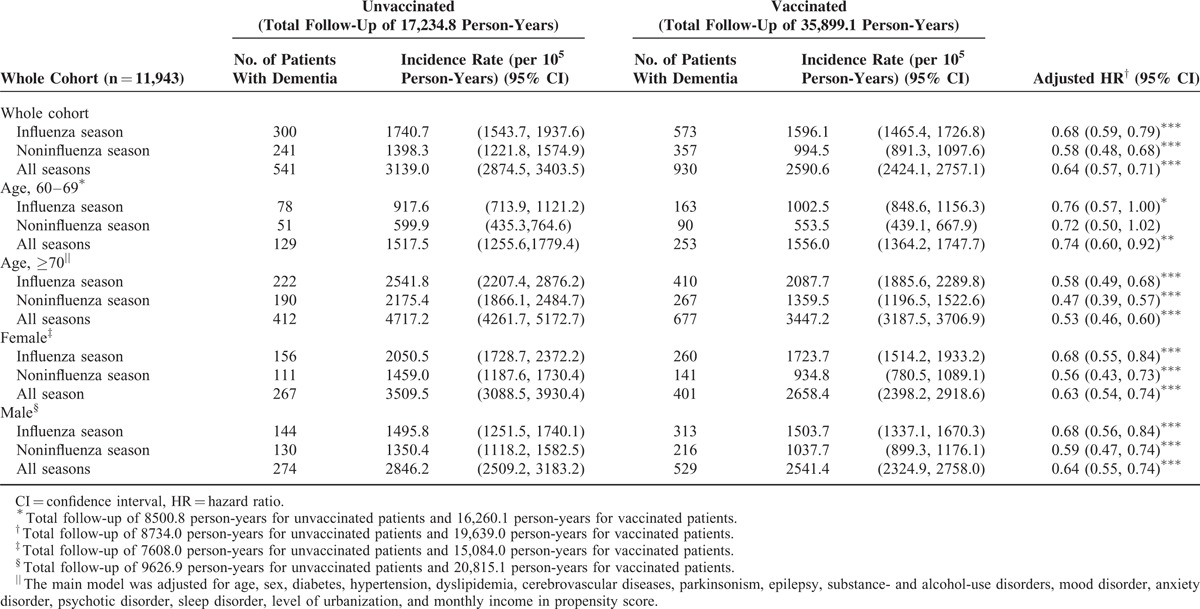
Risk of Dementia Among Unvaccinated and Vaccinated Patients in the Study Cohort

In the sensitivity analysis, adjustments were made to estimate the association of age and sex; diabetes, dyslipidemia, hypertension, cerebrovascular diseases, and anxiety disorder; and statin, metformin, ACEI, and aspirin use with the incidence of dementia in different models. Table 3 shows that the effects of vaccination remained significant in the subgroups of various covariates during the influenza season. Vaccination dose-dependently reduced the risk of dementia in all subgroups and the main model with additional covariates (statin, metformin, ACEI, or aspirin use). All aHRs indicated that vaccination induced >4-fold statistically significant reductions in the risk of dementia in all subgroups, regardless of comorbidities or drug use (*P* < 0.001). Our data revealed that the vaccination frequency reflected the protective effect against dementia during the influenza season. The protective effect was more predominant in patients aged ≥70 years (≥4 vaccinations: aHR = 0.29, 95% confidence interval [CI]: 0.23, 0.33) and in those with diabetes (≥4 vaccinations: aHR = 0.30, 95% CI: 0.22, 0.41) and dyslipidemia (≥4 vaccinations: aHR = 0.31, 95% CI: 0.23, 0.42). As shown in Table 4, we analyzed the sensitivity analysis adjustments in the noninfluenza season. A stronger protective effect against dementia was demonstrated during the noninfluenza season. Less frequent vaccination statistically significantly reduced dementia. Vaccination at a frequency of 2 to 3 times conferred a protective effect to CKD patients. The protective effect was more predominant in patients aged ≥70 years (2–3 vaccinations: aHR = 0.56, 95% CI: 0.43, 0.72; ≥4 vaccinations: aHR = 0.27, 95% CI: 0.21, 0.35) and in those with diabetes (2–3 vaccinations: aHR = 0.58, 95% CI: 0.42, 0.80; ≥4 vaccinations: aHR = 0.25, 95% CI: 0.17, 0.38), dyslipidemia (2–3 vaccinations: aHR = 0.53, 95% CI: 0.37, 0.76; ≥4 vaccinations: aHR = 0.31, 95% CI: 0.21, 0.45), hypertension (2–3 vaccinations: aHR = 0.58, 95% CI: 0.46, 0.75; ≥4 vaccinations: aHR = 0.30, 95% CI: 0.23, 0.39), and cardiovascular diseases (2–3 vaccinations: aHR = 0.59, 95% CI: 0.42, 0.84; ≥4 vaccinations: aHR = 0.29, 95% CI: 0.19, 0.44). During all seasons (Table 5), the trend of dementia reduction still reflected the frequency of vaccination. The protective effect was more predominant in patients aged ≥70 years (2–3 vaccinations: aHR = 0.69, 95% CI: 0.59, 0.80; ≥4 vaccinations: aHR = 0.28, 95% CI: 0.23, 0.33) and in those with diabetes (2–3 vaccinations: aHR = 0.74, 95% CI: 0.61, 0.90; ≥4 vaccinations: aHR = 0.28, 95% CI: 0.22, 0.36), dyslipidemia (2–3 vaccinations: aHR = 0.66, 95% CI: 0.54, 0.82; ≥4 vaccinations: aHR = 0.31, 95% CI: 0.25, 0.39), hypertension (2–3 vaccinations: aHR = 0.74, 95% CI: 0.63, 0.86; ≥4 vaccinations: aHR = 0.34, 95% CI: 0.29, 0.40), and cardiovascular diseases (2–3 vaccinations: aHR = 0.77, 95% CI: 0.62, 0.96; ≥4 vaccinations: aHR = 0.29, 95% CI: 0.33, 0.44). Regardless of statin, metformin, ACEI, or aspirin use, vaccination was an independent protective factor and dose-dependently reduced dementia in patients with CKD.

**TABLE 3:**
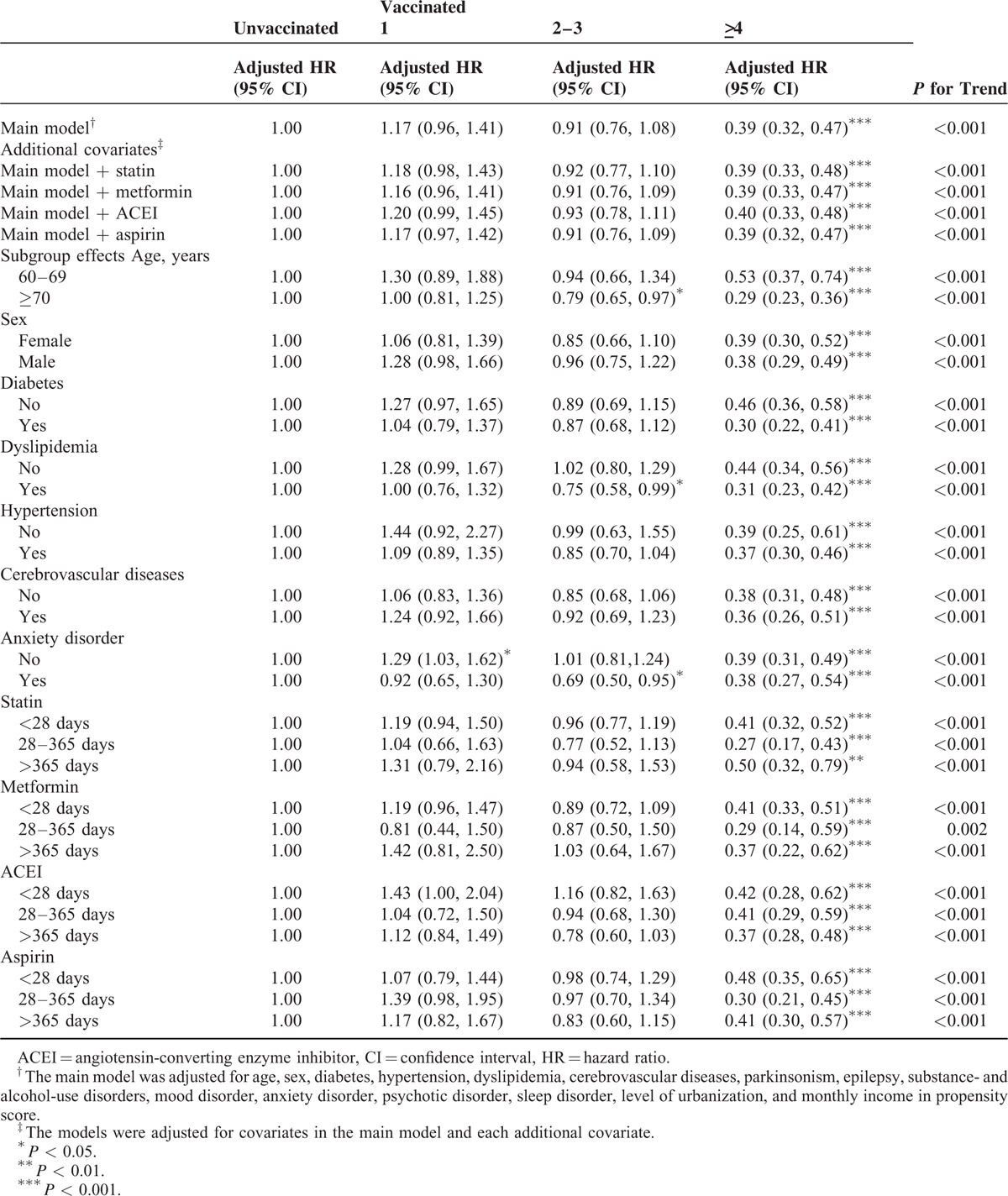
Sensitivity Analysis of Adjusted HRs of Vaccination in Risk Reduction of Dementia in Influenza Season

**TABLE 4:**
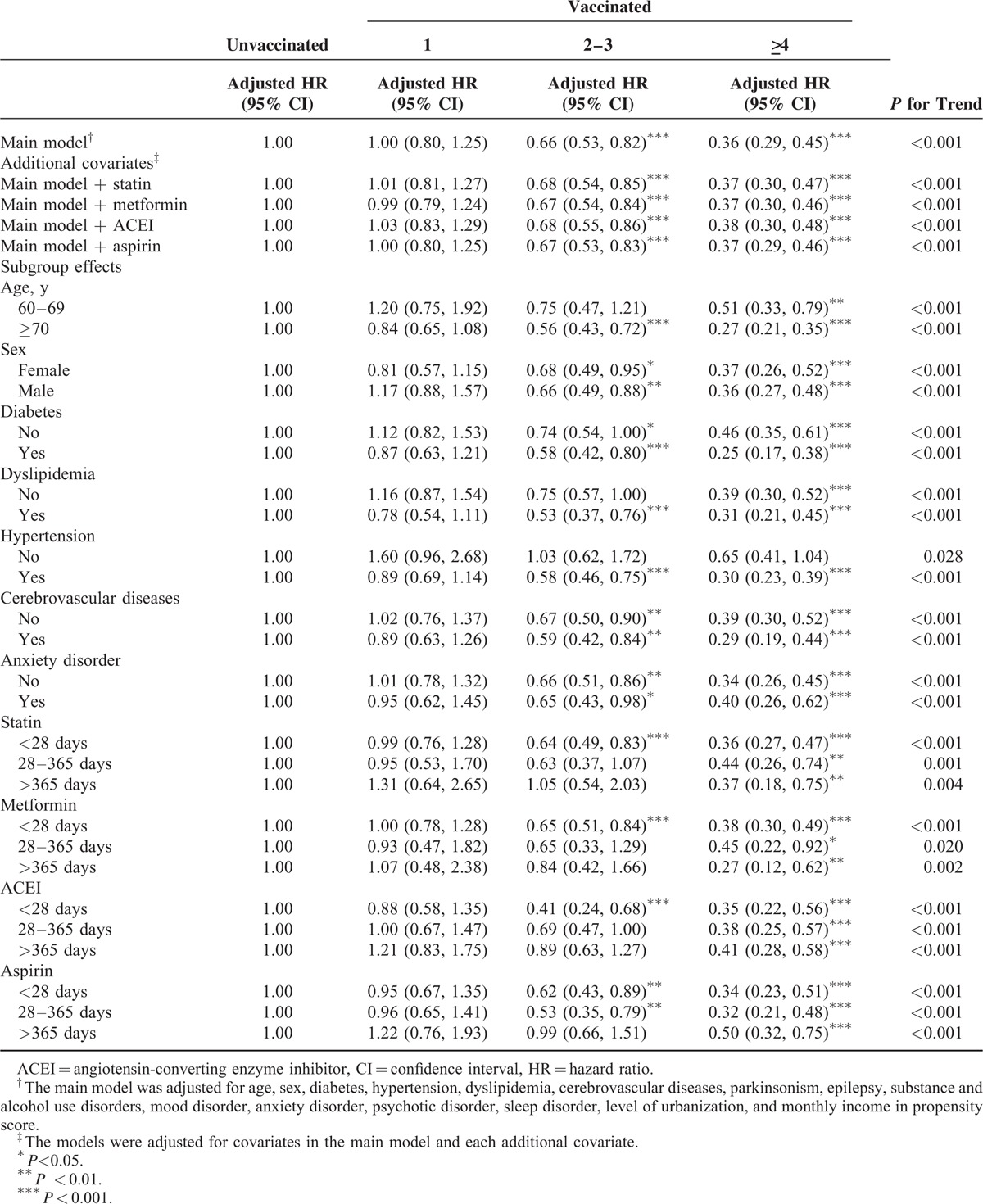
Sensitivity Analysis of Adjusted HRs of Vaccination in Risk Reduction of Dementia in Noninfluenza Season

**TABLE 5:**
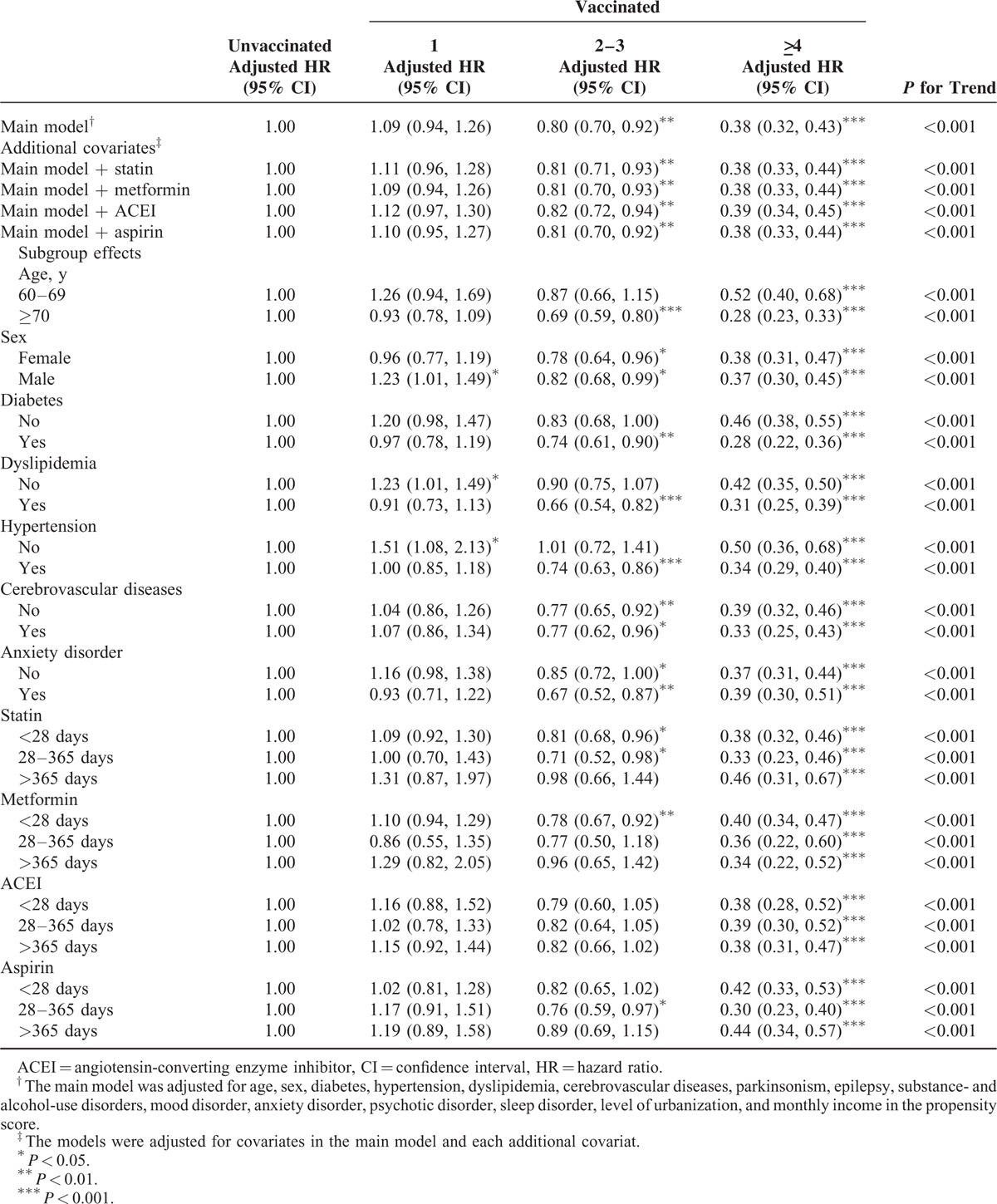
Sensitivity Analysis of Adjusted HRs of Vaccination in Risk Reduction of Dementia in All Seasons

## DISCUSSION

To date, few studies have investigated the association between dementia risk and influenza vaccination. Nichol et al demonstrated that the influenza vaccination of elderly patients is associated with reduced risks of heart and cerebrovascular diseases.^18^ Possible mechanisms of the increased risk of cerebrovascular and cardiovascular events after upper respiratory tract illnesses (e.g., influenza) include alterations in circulating clotting factors, inflammatory response protein levels, and cytokine levels and platelet aggregation and lysis. These changes might enhance thrombotic tendencies, impair vasodilation, or cause endothelial injury. Clinical manifestations of cardiovascular diseases and markers of atherosclerotic disease burden may also be used to identify people at risk for cognitive decline and dementia.^19–22^ Verreault et al showed that previous influenza vaccination may protect against Alzheimer disease (AD) development.^23^ Cerebrovascular diseases and AD frequently coexist. Hypertension is the primary risk factor for vascular diseases in the brain. Cerebrovascular diseases are associated with deteriorated cognitive performance in AD patients, and clinicopathologic studies have suggested that cerebrovascular diseases lower the threshold for clinical dementia in patients with a neuropathologic diagnosis of AD.^24,25^ Our finding that influenza vaccination reduced dementia risk might reflect the response of heart disease, cerebrovascular diseases, and AD decreasing. Based on our literature review, this is the first study to demonstrate the association of dementia risk with influenza vaccination in patients with CKD.

A moderate reduction in GFR is associated with accelerated cognitive decline and increased dementia risk in elderly patients.^5,6^ Among people with good to excellent health, renal impairment is associated with vascular dementia, but not AD. Faster GFR decline is also associated with more significant cognitive decline.^7^ Our observations are consistent with accumulated data showing an increase in the risk of coronary heart disease in patients with mild to moderate CKD; hence, CKD is considered a manifestation of vascular diseases.^26–28^ Vascular risk factors such as hypertension, dyslipidemia, and diabetes are associated with cognitive decline and all-cause dementia and AD and vascular dementia individually. Influenza vaccination reduces the risk of cerebrovascular and cardiovascular diseases.^18^ Vaccination predominately reduced dementia in patients aged ≥70 years (2–3 vaccinations: aHR = 0.69, 95% CI: 0.59, 0.80; ≥4 vaccinations: aHR = 0.28, 95% CI: 0.23, 0.33) and in those with diabetes (2–3 vaccinations: aHR = 0.74, 95% CI: 0.61, 0.90; ≥4 vaccinations: aHR = 0.28, 95% CI: 0.22, 0.36), dyslipidemia (2–3 vaccinations: aHR = 0.66, 95% CI: 0.54, 0.82; ≥4 vaccinations: aHR = 0.31, 95% CI: 0.25, 0.39), hypertension (2–3 vaccinations: aHR = 0.74, 95% CI: 0.63, 0.86; ≥4 vaccinations: aHR = 0.34, 95% CI: 0.29, 0.40), and cardiovascular diseases (2–3 vaccinations: aHR = 0.77, 95% CI: 0.62, 0.96; ≥4 vaccinations: aHR = 0.29, 95% CI: 0.33, 0.44). Our results suggest that influenza vaccination reduces dementia risk in patients with CKD.

Patients with CKD exhibit an increased risk of infection.^29,30^ The 2012 Kidney Disease: Improving Global Outcomes (KDIGO) guidelines recommend that patients with all stages of CKD should be annually vaccinated against influenza, unless contraindicated.^31^ However, whether influenza vaccination reduces the morbidity or mortality of CKD patients is unclear. Studies have suggested that vaccination is associated with a diminished incidence of influenza and all-cause hospitalization, improving the survival of such patients.^32,33^ However, these results may have been confounded by unmeasured prognostic variables. In addition, a study revealed that the antibody response to the influenza vaccine in CKD patients was lower than that in the general population.^34^ This response, in contrast to that to other vaccines, was not significantly different from that in controls, although the number of CKD patients investigated was relatively low. In our study, the largest current series of CKD patients were analyzed, and the protective effect against dementia was obvious. The influenza vaccination of patients with CKD reduced not only mortality (controversial)^33^ but also dementia.

In clinical practice, we suggest that CKD patients with high dementia risk be vaccinated.^5–7^ Whitmer et al identified that hypertension, high cholesterol, and diabetes at midlife were associated with a 20% to 40% increase in dementia risk; multiple risk factors were dose-dependently associated with a higher risk of dementia.^35^ In our study, dementia risk reduction and the protective effect were more predominant in patients with diabetes, dyslipidemia, hypertension, and cerebrovascular diseases compared with other patients. This is because the presence of additional dementia risk factors could proportionally increase the incidence of dementia in patients with CKD; hence, the protective effect of influenza vaccination was stronger. CKD is considered a manifestation of vascular diseases.^26–28^ CKD patients with additional risk factors are more susceptible to dementia. Our findings demonstrate that influenza vaccination had dose-dependent protective effects on elderly CKD patients, particularly those with diabetes, dyslipidemia, hypertension, and cerebrovascular diseases (Tables 3–5).

In this study, the magnitude of the bias demonstrated by the associations observed during the noninfluenza season was sufficient to completely account for the associations observed during the influenza season. A competing risk factor exists between influenza and noninfluenza seasons. Studies have demonstrated an increased risk of hospital admission and a high mortality rate among elderly men during the influenza season.^33,36,37^ The higher mortality risk during the influenza season might mask the incidence of dementia episodes, and CKD patients might die before dementia development. This is why influenza vaccination had a stronger protective effect against dementia during the noninfluenza season (Tables 3 and 4).

Based on our research, this is the first study to demonstrate the association of influenza vaccination with the risk of dementia in CKD patients. Previous studies showed that patients with CKD had a high risk of influenza-related mortality and high risk of dementia; however, we do not have sufficient evidence to resolve the problem in patients with CKD. Our findings demonstrate that the protective effect against dementia risk in patients with CKD was dose dependent. The major solution might be the regular influenza vaccination of CKD patients, particularly those with dementia risk factors such as hypertension, dyslipidemia, diabetes, and cerebrovascular diseases. Tables 3–5 show the sensitivity analysis of the aHRs of age, sex, comorbidities, urbanization level, and monthly income in the PS. The models were adjusted for covariates in the main model and each additional covariate to estimate the reduction in dementia risk during the follow-up period. The dose-dependent protective effect of influenza vaccination was observed regardless of age and sex; diabetes, hypertension, dyslipidemia, cerebrovascular diseases, and anxiety disorder; or ACEI, metformin, statin, and aspirin use, with stratification by various frequencies of influenza vaccination. The dose-dependent protective effect of influenza vaccination was observed for different conditional statuses. In addition, this is the first study to evaluate the dose–response effect of influenza vaccination on dementia risk. Our results show that influenza vaccination was less effective in reducing dementia only once. Influenza vaccines should be administered at least once or >2 to 3 times for achieving a significant protective effect in CKD patients with dementia risk factors during the noninfluenza season (Table 4). The higher the frequency of influenza vaccination is, the more significant the protective effect against dementia in CKD patients becomes. The strength of the present study is its large sample size. The results suggest that the incidence of dementia was reduced in CKD patients through the preventive strategy of influenza vaccination. This is the first study to demonstrate that influenza vaccination exerts dose–response and synergistic protective effects against dementia in CKD patients with dementia risk factors by reducing the incidence of dementia.

This study has potential limitations. Evidence from observational studies suggests that lifestyle factors, particularly social, mental, and physical activity, are inversely associated with the risk of cognitive decline and dementia. However, methodologic concerns may obscure the precise relationship between these factors and dementia risk. One theory states that higher educational levels and cognitive activity produce a cognitive reserve that reduces the impact of neurodegeneration on cognitive function. In our study, we used the PS to match age, sex, comorbidities, urbanization level, and monthly income. The urbanization level and monthly income are unvalidated alternatives to lifestyle factors and the educational level. To obtain such information, a large randomized trial should apply a suitable regimen to appropriately selected patients for comparing standard approaches. Moreover, the diagnoses of dementia and all other comorbidities were completely dependent on the ICD codes. Nevertheless, the National Health Insurance Administration randomly reviews medical records and interviews patients to validate diagnoses. Hospitals with outlier diagnoses and practices may be audited and subsequently heavily penalized if malpractice and discrepancies are discovered. Another limitation is that information on several unmeasured confounders, including body mass index, smoking, alcohol intake, and use of other over-the-counter drugs (which are associated with dementia), is not available in the NHIRD. However, considering the magnitude and significance of the observed effects, it is unlikely that these limitations compromised the results. Finally, our study is not a prospective randomized blinded study; hence, a cause–effect relationship could not be established. The findings of this study suggest that influenza vaccination exerts a significant protective effect against dementia in CKD patients with dementia risk factors by reducing the incidence of dementia. Additional randomized studies are required to verify these findings.

## CONCLUSIONS

Influenza vaccination exerts dose–response and synergistic protective effects against dementia in CKD patients with dementia risk factors by reducing the incidence of dementia.
